# The role of reservoir species in mediating plague's dynamic response to climate

**DOI:** 10.1098/rsos.230021

**Published:** 2023-05-17

**Authors:** Henry Gillies Fell, Matthew Jones, Steve Atkinson, Nils Christian Stenseth, Adam C. Algar

**Affiliations:** ^1^ School of Geography, University of Nottingham, Nottingham NG7 2RD, UK; ^2^ Centre for Biomolecular Sciences, Nottingham University, Nottingham NG7 2JE, UK; ^3^ Department of Biosciences, Centre for Ecological and Evolutionary Synthesis, University of Oslo, Oslo 0316, Norway; ^4^ Department of Earth System Science, Ministry of Education Key Laboratory for Earth System Modeling, Tsinghua University, Beijing 100084, China; ^5^ Department of Biology, Lakehead University, Ontario P7B 5E1, Canada

**Keywords:** plague, *Yersinia pestis*, third pandemic, environmental niche modelling, hindcasting

## Abstract

The distribution and transmission of *Yersinia pestis*, the bacterial agent of plague, responds dynamically to climate, both within wildlife reservoirs and human populations. The exact mechanisms mediating plague's response to climate are still poorly understood, particularly across large environmentally heterogeneous regions encompassing several reservoir species. A heterogeneous response to precipitation was observed in plague intensity across northern and southern China during the Third Pandemic. This has been attributed to the response of reservoir species in each region. We use environmental niche modelling and hindcasting methods to test the response of a broad range of reservoir species to precipitation. We find little support for the hypothesis that the response of reservoir species to precipitation mediated the impact of precipitation on plague intensity. We instead observed that precipitation variables were of limited importance in defining species niches and rarely showed the expected response to precipitation across northern and southern China. These findings do not suggest that precipitation–reservoir species dynamics never influence plague intensity but that instead, the response of reservoir species to precipitation across a single biome cannot be assumed and that limited numbers of reservoir species may have a disproportional impact upon plague intensity.

## 1. Introduction

The distribution and dissemination of the plague bacterium *Yersinia pestis* is linked to climate dynamics [1] across a range of biological scales [2–5]. However, the response to climate is far from homogeneous, given the wide range of reservoir and vector species, and environments in which *Y. pestis* persists [6–10]. Along with vectors and bacteria, the dynamics of reservoir species are key elements in understanding the mechanisms which drive epidemic and enzootic cycles [6,11,12]. Inferences are often made about the ecological mechanisms that link climate to the transmission of plague within human populations, based on the response of one, or a few, reservoir species which may not be representative of the entire, complex, multi-reservoir species system [13]. Here, we focus on how differential responses of reservoir species to precipitation may mediate geographical variation in climate–plague dynamics. Specifically, we test the hypothesis that plague's heterogeneous response to climate (precipitation) across China during the Third Pandemic (1772 C.E–1964 CE) was driven by consistent differences in how individual reservoir species responded to changing precipitation [7].

The Third Pandemic caused fewer fatalities than the previous two; however, it led to the establishment of plague reservoirs in North and South America and therefore drove the current global distribution of *Y. pestis* [7,14]. The Third Pandemic began in Yunnan province in 1772, where spread was gradual. Cases across Yunnan were limited but persisted and led to eventual transmission to the rest of southern China, where cases began to increase from 1850 [15]. *Yersinia pestis* entered Hong Kong in 1894 and was subsequently transmitted globally through international shipping routes at the turn of the twentieth century, which also coincided with the peak in cases across China [15]. The epidemic persisted in China until the start of the plague control programme around 1950, which was preceded by a substantial final peak in recorded cases [14,15].

During the Third Pandemic, plague intensity (proportion of human population infected) in China responded heterogeneously to climatic variation [7]. Plague reservoir species are predominantly, although not solely, rodent species which are known to host the *Y. pestis* bacteria with inter- and intra-species transmission driven by a broad range of vector (flea) species. In northern China, the reservoir species include several marmot (e.g. *Marmota caudata*), gerbil (e.g. *Meriones unguiculatus*) and ground squirrel (e.g. *Spermophilus dauricus*), whereas in southern China the reservoir species are more commonly rat (e.g. *Rattus rattus* and *Rattus tanezumi*) [8]. North China has a predominantly arid to semi-arid environment, conditions which are shared by many *Y. pestis* reservoir species and their vectors [16]. Plague intensity generally increased with wetness in the preceding year and followed a hump-shaped relationship with contemporary (unlagged) wetness [7]. By contrast, South China has high annual precipitation due to the South Asian Monsoon [17]. In this region, plague intensity generally increased with the preceding year's dryness and showed a U-shaped relationship with contemporary dryness [7].

Xu *et al.* [7] hypothesized that geographical variation in plague's response to climate during the Third Pandemic reflected differential responses of reservoir species to precipitation between regions. In the North, they proposed a bottom-up trophic cascade, a mechanism previously posited as a driver of plague epizootic cycles in several regions [1,5,11,18]. This hypothesis proposes that reservoir and vector species populations are positively impacted by increased precipitation via increased primary productivity, leading to greater food resources for reservoir species within precipitation-limited environments [11,18]. Xu *et al.* [7] further suggested that the cascade collapses when precipitation is extremely high, where the reservoir populations may decline due to increased mortality through the flooding of burrows. The trophic cascade mechanism proposed for North China is not consistent with South China where dryness correlates positively with plague intensity. The exact mechanism driving this correlation is still unknown but several observations suggest that reservoir species respond differently to wetness than in the North. For example, *Rattus* spp*.* which are more common in the South, are human commensal rodents whose population sizes correlate negatively with precipitation in this region [7]. Other southern reservoir species, such as *Microtus fortis,* live on river and lake banks and are therefore highly sensitive to precipitation due to changing water levels, which may partially contribute to the observed plague–dryness relationship [7,19].

Vector species are also sensitive to climatic conditions, particularly on a microclimatic scale, such as within burrow environments or the soil substrate [20]. Sufficient numbers of active fleas within the reservoir-vector system are required for *Y. pestis* transmission [21]. Vector species' response to climate should therefore also be investigated.

Testing whether reservoir and vector species in North and South China show systematic differences in their responses to precipitation requires long-term time series on population size for multiple reservoir species. Unless significant long-term sampling efforts have been used (see plague literature in the Balkhash region of Kazakhstan [22]), these data are rarely available. In the absence of such data, environmental niche models (ENMs) can provide information on the suitability of environmental conditions for a species, and in many cases, are a reasonable proxy for abundance, though such relationships are far from perfect and this may vary from species to species [23,24]. These models can be built with modern data and then hindcast (projected into past climatic space) to estimate responses of species to past climatic change [25]. Here, we use ENMs and hindcasting to test the hypothesis that plague's heterogeneous response to precipitation across China is driven by reservoir species' responses to precipitation.

Specifically, we make three inter-related predictions. First, we predict that precipitation variables will make large contributions to fit of each species ENM variables (Prediction 1a), with a positive relationship between niche suitability and precipitation variables in the North and the opposite in the South (Prediction 1b). Second, that the species niche suitability would positively correlate with precipitation for northern species through time and negatively for southern species (Prediction 2). Third, we predicted that the niche suitability of northern species would increase during wet periods, while for southern species, the niche suitability would increase during dry periods (Prediction 3). While the outcomes of Predictions 2 and 3 are strongly related to Prediction 1, differences in the magnitude, and the interaction of non-precipitation climatic variables through time could decouple overall changes in niche suitability from changes in precipitation. Predictions 2 and 3 therefore act as a test of Prediction 1 in time and space.

## 2. Materials and methods

### 2.1. Reservoir species localities

We selected 38 putative reservoir mammal species (electronic supplementary material, table S1) across China based on Mahmoudi *et al.* [8] and Cui *et al.* [26]. Occurrences for reservoir species were sourced from the Global Biodiversity Information Facility (GBIF, electronic supplementary material, table S1) using the *rgbif* package [27]. We clipped occurrences to a broad Eurasian extent (10 W, 150 E, 0 S, 80 N) to avoid imposing bias in niche estimates by limiting the available climate space to China only [28] and removed occurrences with probably erroneous coordinates (electronic supplementary material, table S1). We thinned each species’ occurrences to a maximum of one occurrence per cell of climate data (0.5° resolution, see below) using the elimCellDups function in the *enmSdm* package [29,30]. We then excluded species with 10 or fewer occurrence points, leaving 33 species (electronic supplementary material, table S1). We further selected 38 *Y. pestis* vector species associated with the selected reservoir species across Eurasia; however, only one species (*Ctenocephalides felis*) had greater than 10 occurrences. Analysis of this species is included in the electronic supplementary material, along with the complete list of vector species (electronic supplementary material, tables S2 and S3).

### 2.2. Historical climate data

We obtained historical climate data (mean, maximum and minimum daily temperature and total daily precipitation) from the ISIMIP2b project, which provides modelled data from 1661 to 2005 [25]. We repeated all analyses with data from two global climate models (GCMs): (i) the National Oceanic and Atmospheric Administration geophysical fluid dynamics laboratory GCM, henceforth GFDL, and (ii) the Institut Pierre-Simon Laplace GCM, henceforth IPSL [31]. These two models capture a range of possible past climate conditions as is recommended when hindcasting [25]. We calculated monthly averages from daily values and then used the biovar function of the dismo package [32] to calculate the 19 bioclimate variables used by the WorldClim dataset and which are commonly used for environmental niche modelling [33].

### 2.3. Niche modelling and hindcasting

We fit ENMs for each reservoir species individually using Maxent [34], implemented using the enmevaluate function of the ENMeval package [35]. We used bioclimate variables averaged from 1950 to 2000 (recent climate hereafter) as predictors. Although dynamic ENMs using climate date matched to species occurrences were trialled, this proved inconclusive across the models and was therefore not used. This, however, is an area which requires further research, particularly in studying climatically dynamic disease distributions [36]. Annual precipitation variables from both model scenarios used here are highly correlated (correlation 0.91, *p* < 0.001) with the Worldclim annual precipitation data [33] across all species occurrence points, helping to validate the use of these data for model construction. Full details on variable selection, temporal distribution of occurrences, model tuning and internal validation for each species are provided in the electronic supplementary material, methods. All species with an area under the curve (AUC) of the receiver operating characteristic of less than 0.7 across the China plague extent (CPE, 71.2 E, 135.1 E, 15.4 N, 54.6 N) were removed from further analysis. ENMs constructed for each species using recent climate were hindcast into annual historical climate space using clamped and unclamped projections [37] for both climate scenarios (IPSL and GFDL). The models were constructed over the Eurasian extent and then projected to the CPE.

### 2.4. Hypothesis testing

#### 2.4.1. Response curves and variable importance (Prediction 1)

**Figure 1.**
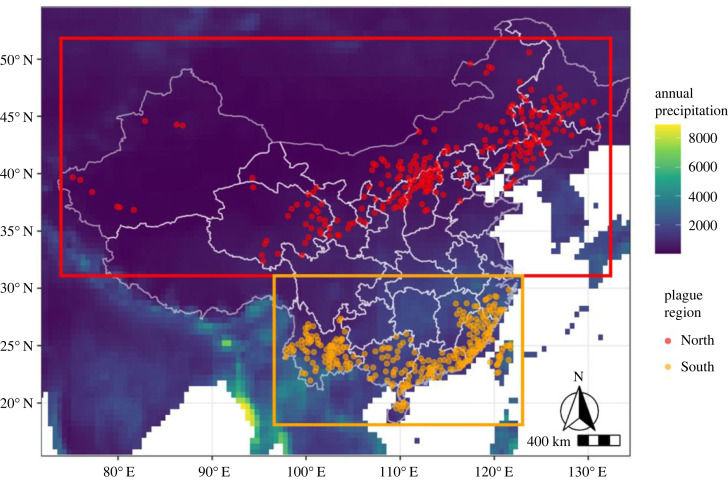
Distribution of human plague cases across China during the Third Pandemic coloured by region (north red, south orange) with the mean annual precipitation between 1950 and 2000 and Chinese regional borders (white lines) plotted [7,38].

Following Xu *et al.* [7], we used human plague data to define the northern and southern regions of China (figure 1), with the divide at 31°N. To determine whether reservoir species responded strongly and positively (north) or negatively (south) to precipitation (Predictions 1a and 1b), we computed variable response curves and permutation importance for each variable from the recent ENM for each species [37]. We considered results consistent with the hypothesis if a precipitation variable had the highest or second-highest permutation importance, and the response curve was in the predicted direction.

#### 2.4.2. Niche suitability time series (Prediction 2)

We generated annual time series in niche suitability for each species by summing suitability scores across all map grid cells in each region for each year from 1661 to 2005. We used two different methods when summing niche suitability, an unrestricted method where all grid cells were included and a restricted method where only those grid cells that fell within a buffered minimum convex polygon (BMCP) based on a species' occurrences across all years were included [39,40]. Buffer distance around the MCP area was based on the maximum distance of minimal pairwise distances between occurrences [39]. We standardized each species’ time series using a *z*-score transformation and computed the correlation between mean annual precipitation across the region in which the species is found and suitability through time. We calculated mean correlation values across each species, region and climate scenario both individually and combined across the regions and climate scenarios (table 1; electronic supplementary material, tables S4–S6). The correlation statistics were calculated with no lag and a 1-year lag period.

**Table 1.** Correlation of reservoir species niche suitability values through time (summed) and annual precipitation values. Correlation was compared across two climate scenarios, each with clamped and unclamped model versions. Reds represent the lowest (most negative values) and blues the highest (most positive values) correlations. Bold values are statistically significant (*p* < 0.05) or are calculated with statistically significant values (mean and s.d.).

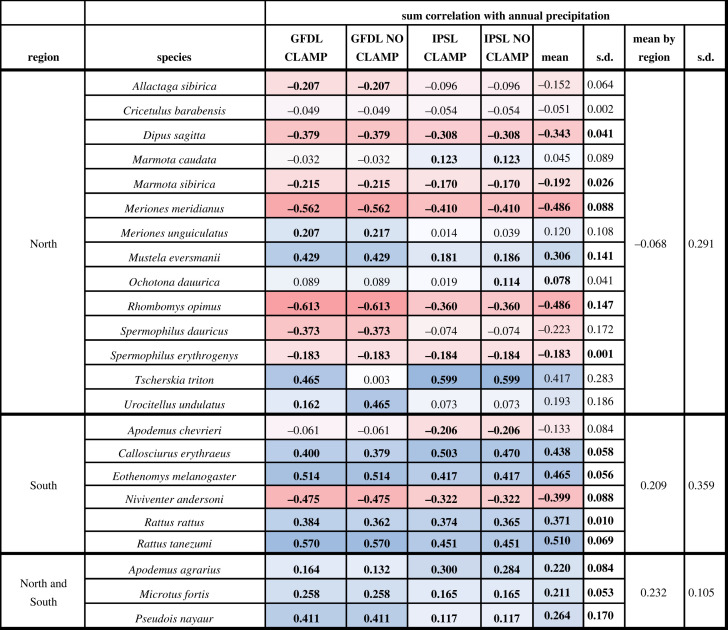

#### 2.4.3. Wet and dry periods (Prediction 3)

**Figure 2.**
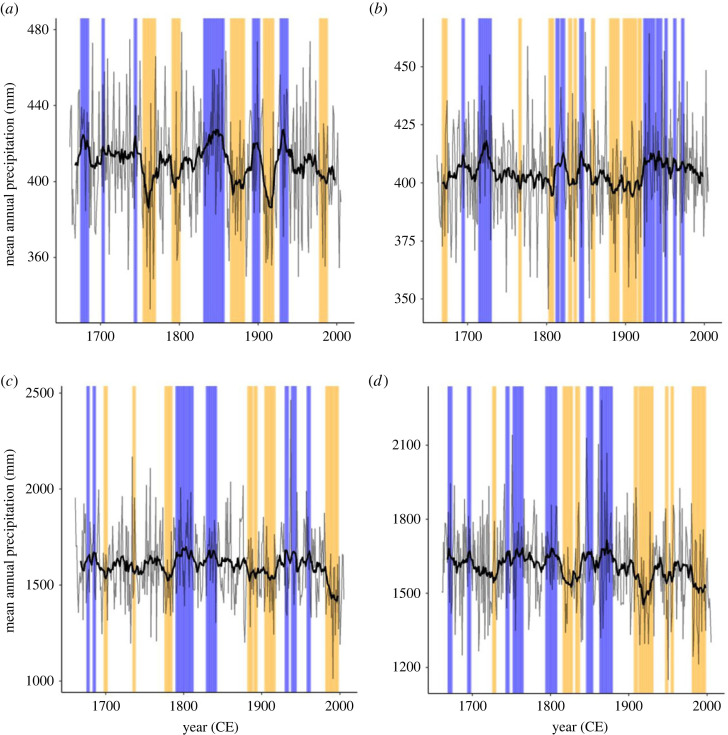
Identification of wet and dry periods in the ISIMIP data across both climate scenarios. (*a*) North China GFDL scenario, (*b*) North China IPSL, (*c*) South China GFDL and (*d*) South China IPSL. Blue indicates the wettest quartile and orange indicates the driest quartile. Both annual (thin black line) and 15-year running average (thick black line) mean annual precipitation are shown.

We identified wet and dry periods in each region by calculating a 15-year running average of precipitation from 1661 to 2005 and delineated the upper and lower quartiles of this data as wet and dry periods respectively (figure 2). Periods of less than 3 consecutive years were excluded. We then calculated the mean suitability in each wet or dry period from the standardized niche suitability for each species across clamped and unclamped projections. We predicted a positive mean niche suitability in regions and conditions where reservoir species were suggested to catalyse an increase in plague intensity (northern wet periods, southern dry periods).

## 3. Results

### 3.1. Niche modelling

#### 3.1.1. Model performance

ENMs fit using buffered Eurasian ranges (species extents) fit reasonably well; the mean AUC across all climate scenarios and clamping methods was 0.817 (s.d.: 0.074, electronic supplementary material, table S7). All but two species, *Niviventer andersoni* and *Rhombomys opimus* had AUCs > 0.7. ENMs fitted using the CPE study region had, on average, lower and variable AUCs (mean: 0.78, s.d. = 0.19, table 1) with 10 species having AUC < 0.7. As all further analysis was completed over the CPE, the 10 species with an AUC lower than 0.7 were subsequently removed. Seventy per cent of species had a mean difference between training and test AUC (a measure of overfitting) of less than 0.1, suggesting limited overfitting of models (electronic supplementary material, table S8) [41].

### 3.2. Hypothesis testing

#### 3.2.1. Response curves

**Figure 3.**
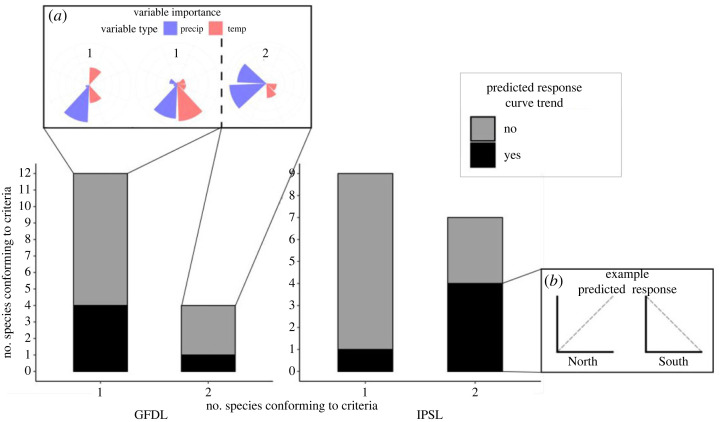
Staked bar chart illustrating the number species unclamped models which conform to the response (prediction 1a) and variable importance predictions (prediction 1b). The *y*-axis represents the number of species conforming to the variable importance criteria. The *x*-axis represents how many variables within each species model conform to the importance criteria, with inset box (*a*) showing the differing variable importance requirements to conform to prediction 1a. Species which further conform to the response curve criteria (1b) are coloured black and non-conforming species are coloured grey. Inset box (*b*) shows a linear example of the expected response in each region.

Across all scenarios, a minority of species conformed to the response and variable importance prediction criteria (five across each scenario) (figure 3; electronic supplementary material, figure S1). There was no variation in the number of species which conformed across both climate scenario or clamping methods. However, species which conformed to the predictions varied across clamping methods and climate scenarios with five species showing consistency across three of the four scenarios (climate and clamping method). Selected importance and response curve plots are presented in electronic supplementary material, figure S2. The one included vector species had a precipitation variable as one of the two most important variables, but as the species extent covered both regions the response curves were not informative with regard to the impact of precipitation on the northern region (electronic supplementary material, table S3).

#### 3.2.2. Niche time-series correlation

Across all regions, species showed a range of responses to changes in precipitation through time, regardless of climate scenario, clamping, or whether unlagged or 1-year lagged precipitation data were considered. In the North, 6 of 14 species (43%) showed the predicted positive correlation between summed unlagged niche suitability; however, only two of these species (*Mustela eversmanii* and *Ochotona dauurica)* were statistically significant (*p* < 0.05) across all scenarios (table 1). Mean correlations varied from −0.486 to 0.417 with an overall mean of −0.068 (s.d. = 0.291) across all northern species. Although the magnitude of the correlation varied across climate and clamping scenarios within northern species, the direction was consistent for all northern species but one (*Marmota caudata,* table 1). In the South, two species had the predicted negative correlation with annual precipitation, *Apodemus chevrieri* and *N. andersoni*, the correlation of the latter however was not statistically significant (*p* < 0.05). The remainder of the southern species (four) had positive mean correlations with precipitation through time (*r* = 0.371–0.510; table 1). Lastly, species classified as both northern and southern (three species), either due to a broad Eurasian extent or distribution in central China, all showed a positive correlation with precipitation (table 1). The results for the spatially constrained (BMCP) niche models for each species were generally consistent with the unconstrained models, though the direction of the correlation changed for two species: *A. chevrieri and M. caudata*, neither of which were statistically significant (*p* < 0.05) (electronic supplementary material, table S4). Following Xu *et al.* [7], we repeated the correlation analysis using climate data from the year prior (electronic supplementary material, tables S5 and S6). Generally, all correlations were weaker in the lagged version and the direction of correlation was maintained for most species. In the cases where a change in correlation direction occurred, (*O. dauurica, Urocitellus undulatus, A. agrarius* and *M. fortis*), the correlation was consistently weak (|*r*| < 0.1). The included vector species showed a high and significant (*p* < 0.05) correlation with precipitation when unlagged and a similarly muted correlation when lagged (electronic supplementary material, table S3).

#### 3.2.3. Wet and dry period niche analysis

During northern wet periods, under the GFDL climate scenario, 6 of 14 species (5 of 14 under IPSL) had increased mean niche suitability (table 2). However, only two of these (none under IPSL) had an increased mean niche suitability greater than 0.1 (table 2). As the mean niche suitability values were standardized, this was an increase in mean niche suitability of 0.1 s.d., highlighting the limited variation in mean niche suitability in many species through periods of high precipitation. In northern dry periods, under GFDL, 10 species (eight under IPSL) had an increased mean niche suitability; eight of these had mean values > 0.1 (three for IPSL).

**Table 2.** Mean standardized niche suitability of each reservoir species through wet and dry periods. Mean and s.d. values were calculated across periods identified as wet or dry and a blue–red colour scale is applied with red representing the lowest (most negative values) and blue representing the highest (most positive values). Reservoir species are grouped by region. The mean was calculated across both clamped and unclamped model projections. Mean comparisons between species were qualitative. Bold values show mean suitability's of greater than 0.1 which support prediction 3.

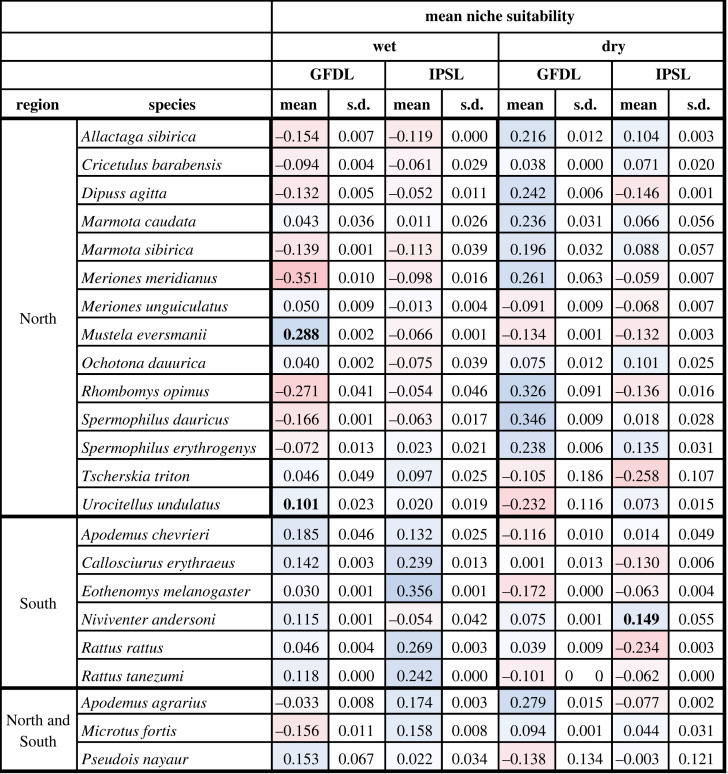

In the southern region during dry periods under GFDL three of six (however, one species suitability = 0.001) species (two of eight = six under IPSL) had increased mean niche suitability, of these none had mean values greater than 0.1 (1 for IPSL). The one species which had a mean greater than 0.1 under IPSL was *N. andersoni*. This species conformed to the hypothesis predictions through each method (Predictions 1, 2 and 3). The observed pattern was reversed during wet periods where, under GFDL, all (six of six) species had a positive mean (five of six under IPSL), of which four had mean values greater than 0.1 (five for IPSL). The clamped and unclamped projections were combined when calculating mean niche suitability and the low mean s.d. across all species (0.024) suggests that there was little variation between the two methods.

## 4. Discussion

We found minimal evidence using an ENM approach consistent with the hypothesis that, in China during the Third Pandemic, the heterogeneous response of plague intensity to precipitation was mediated by geographical differences in how reservoir species responded to precipitation. Instead, we found that precipitation variables were generally of limited importance in ENMs and did not consistently show the predicted relationship with niche suitability. In North China, contrary to predictions, there was no consistent positive correlation between reservoir species' niche suitability and precipitation, while in South China, we predominantly found positive instead of the predicted negative correlations. Also in contrast with our predictions, species niche suitability was found to be no higher during periods of increased precipitation in the North or decreased precipitation in the South; in fact the opposite was observed with niche suitability increasing in the North during dry periods and decreasing in the South during wet periods. These findings indicate that how reservoir species mediate climate–plague relationships is complex and cannot be generalized across a diverse suite of potential reservoir species. Inferences regarding climate–reservoir–plague interactions based on one, or a few, reservoir species are unlikely to be representative of general reservoir responses, and such extrapolations should be avoided. We attempted to complete similar analysis using vector species; however, the limited number of occurrences in GBIF limited the use of our selected method. Given the likely importance in plague–climate relationships, effort should be made to increase plague vector occurrence data through increased sampling efforts or climatic tolerance analysis to enable mechanistic niche modelling methods to be applied to vector species.

Although overall we found no consistent response of reservoir species to precipitation in either the North or South, just under half of the northern species showed the predicted positive correlation between niche suitability and precipitation. This is consistent with the bottom-up trophic cascade mechanism regularly applied to plague systems, where elevated precipitation enhances primary productivity, thereby increasing carrying capacity of reservoir species [1,5,11,18]. The remaining northern species showed a negative correlation, inconsistent with a bottom-up trophic cascade. Other mechanisms may mediate the impact of precipitation on plague intensity through these species. For example, a population decline mediated by climate may increase the vector load per individual reservoir species [3]. This would facilitate increased transmission within the reservoir as well as drive transmission to alternative reservoir species [42]. Alternatively, the dynamics of plague intensity may be decoupled from the population dynamics of some of these potential reservoir species.

In the South, only one species conformed to predictions, with most species showing a positive correlation between precipitation and niche suitability. While this does not match our predictions, it is potentially still consistent with the ‘U’-shaped response to precipitation observed in South China [7] where very wet periods correlate with increased plague intensity. However, this ‘U’-shaped response is difficult to falsify as the opposite response in plague intensity is expected under wet and very wet conditions and the delineations between these conditions are probably spatially heterogeneous. As in the North, our findings suggest a non-homogeneous response from reservoir species to changes in precipitation, and thus, if these species are driving plague transmission, there is unlikely to be a single mechanism operating across the entire range of species.

Very few species conformed to our predictions of high niche suitability during wet periods in the North and during dry periods in the South. In fact, we observed the opposite. The highest niche suitability values were observed under the opposite precipitation regimes than predicted. One possible explanation for these inverse relationships is that they reflect the hump and U-shaped relationships proposed by Xu *et al.* [7] between plague intensity and precipitation. However, we think this explanation unlikely as the delineated wet and dry periods, while at the limits for our dataset, probably underestimate real extreme conditions. Although, across China, the ISIMIP data has been found to simulate extreme continuous multi-day rainfall well [43], the conversion to annual climate variables will have muted these extremes, and thus the identified wet and dry periods are representative of moderate and not extreme precipitation. This suggests the wet–dry periods are useful for testing the general, monotonic trends proposed by Xu *et al.* [7], but not the hump and ‘U’-shaped correlations.

Our results are reliant on the assumption that climatic niche suitability is a reasonable proxy for abundance; however, this is not consistently the case [24]. If this assumption is not correct for the species under investigation, then we can instead attribute the lack of consistent correlation or directional change to a disconnect between climate and abundance. This would, however, suggest that if climate effects on host population sizes were mediating the observed relationship, abundances would need to be highly disconnected from climatic suitability to the extent that they would show opposite relationships, which is highly unlikely.

Our results indicate that, contrary to the existing hypothesis [7], differences in plague intensity–precipitation between North and South China cannot be attributed to general differences in how reservoir species respond to precipitation. Within each region, we found a diversity of reservoir species niche suitability–precipitation relationships, spanning from negative to positive. However, this lack of a consistent response does not mean that precipitation–reservoir population dynamics never influence plague intensity. It may be that a single, or small number of, reservoir species that respond as hypothesized are underlying North–South differences in plague intensity. For example *N. andersoni* (South) responded as predicted across all scenarios, but there is sparse literature on of the role of *N. andersoni* in plague maintenance in South China [8]. There is no direct evidence to suggest that this species was key to infection and transmission during the Third Pandemic or that they disproportionately influenced plague transmission compared with other species. Nevertheless, this example highlights that within any region, at any point in time, there is likely to be at least one, but usually more, reservoir species with the potential to exist at high population densities and to enhance plague intensity in human populations.

Key to furthering our understanding of the climate–reservoir–plague nexus will be to develop reliable predictions of which reservoir species will have disproportionate effects, and to integrate climatic influences alongside further elements of the plague system. Further vector species dynamics must be integrated as the response of vector species to climate may be having a larger influence than mammalian reservoir species. Many of the southern species, particularly *Rattus* spp., are commensal rodents closely associated with human habitation and crops, and thus may be influenced only indirectly by climate through its effect on agricultural lands, e.g. crop failure [44]. Further research into the impact of land cover on such species in the context of zoonotic disease is therefore highly relevant. Lastly, mechanisms independent of reservoir population dynamics such as human movement, transmission and population dynamics may contribute to the heterogeneous response of plague intensity to precipitation [45]. While our models, in their present form, cannot effectively test these alternatives, it is clear that assuming consistent reservoir species dynamics in response to climatic variation is an oversimplification of the highly complex plague transmission system and that mechanisms should not be extrapolated to entire systems based on studies of single, or small numbers, of reservoir species.

## Data Availability

The raw data for this analysis are all freely available. The ISIMIP2b data used are a combination of the pre-industrial control runs (1880–2005) and the historical data (1880–1660), accessible through the ISIMIP Repository (https://data.isimip.org/). The ISIMIP data were transformed into annual biovar variables, these data are available through figshare (http://dx.doi.org/10.6084/m9.figshare.21401460). All host species data was obtained from GBIF (www.gbif.org), the reference DOIs for each species are included in the electronic supplementary material (table S1), and the clipped and thinned data for each species has further been uploaded to figshare (http://dx.doi.org/10.6084/m9.figshare.21401460) along with script and functions for completing the analysis. The data are provided in the electronic supplementary material [46].
